# Unilateral simultaneous renal oncocytoma and angiomyolipoma: case report

**DOI:** 10.1186/1757-1626-2-9093

**Published:** 2009-11-26

**Authors:** Theodosios Theodosopoulos, Anneza Yiallourou, Maria Kyriazi, Georgios Anastasopoulos, Evi Kairi-Vassilatou, Nicolaos Dafnios, Ioannis Vassiliou

**Affiliations:** 1 2nd Department of Surgery, Aretaieion Hospital, University of Athens, Greece; 2 Department of Pathology, Aretaieion Hospital, University of Athens, Greece

## Abstract

A rare case of synchronous angiomyolipoma and oncocytoma in the same kidney of a 70 year old man is presented. A left renal mass was found incidentally by ultrasound. Computerized tomography and magnetic resonance imaging revealed a 1,3 cm mass in the mid-portion of the left kidney, whereas on the lower pole of the same kidney, a 3,3 cm mass was also revealed, consistent with angiomyolipoma. A working diagnosis of renal cell carcinoma was made. A radical nephrectomy was performed. Microscopically, the tumor of the lower pole was found to be an angiomyolipoma, whereas the mid-portion tumor was an oncocytoma. Until now, only 16 cases of unilateral simultaneous presence of renal angiomyolipoma and oncocytoma have been reported. Of these cases, all except one were female and three were associated with the tuberous sclerosis complex. It is well worth remarking, that renal oncocytoma overlap with other renal neoplasms, therefore nephrectomy remains the treatment of choice.

Renal angiomyolipoma and oncocytoma are uncommon neoplasms and their simultaneous presence in the same kidney is rare. Only 16 cases have been reported until now in the literature. The purpose of this paper is to present an additional case without evidence of tuberous sclerosis.

## Introduction

Renal angiomyolipoma and oncocytoma represent uncommon neoplasms and their simultaneous presence in the same kidney is rather rare. To the best of our knowledge, only 16 cases have been reported in the literature [1-3]. Oncocytoma, originating from renal tubular cells, is a relatively recently reported benign epithelial tumor that accounts for about 5% of surgically resected renal neoplasms in adults [4]. The nomenclature of angiomyolipoma was first introduced by Morgan et al in 1951[5] to describe a renal tumor that contained a berrant vasculature with variable amounts of intermixed smooth muscle and adipose tissue. Renal angiomyolipoma, generally of embryonal cell origin, represents less than 1% of all surgically removed tumors and is frequently associated with tuberous sclerosis [6]. Oncocytomas have also been associated with cortical adenomas and renal cell carcinoma, whereas angiomyolipomas have been associated with renal cell carcinomas, a papillary adenoma, a cystic nephroma and a metanephric adenoma [1,7,8].

We present a case report of renal angiomyolipoma and oncocytoma without evidence of tuberous sclerosis.

## Case presentation

### Clinical Case

**Figure 1:**
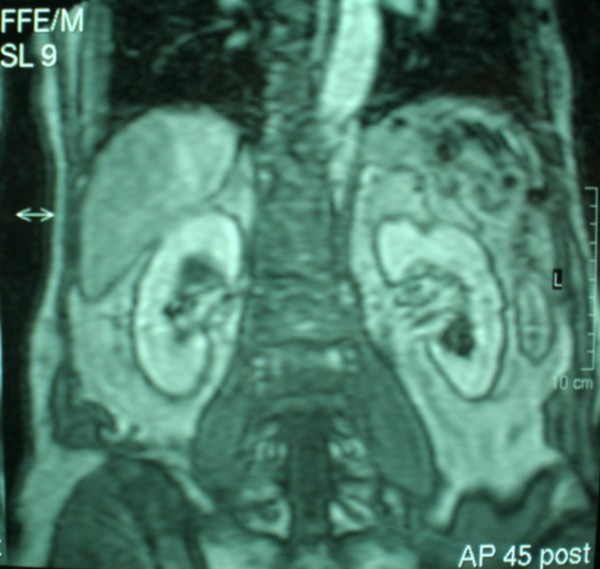
**Magnetic resonance imaging: Well- defined, homogenous mass in the mid- portion of the left kidney**.

**Figure 2:**
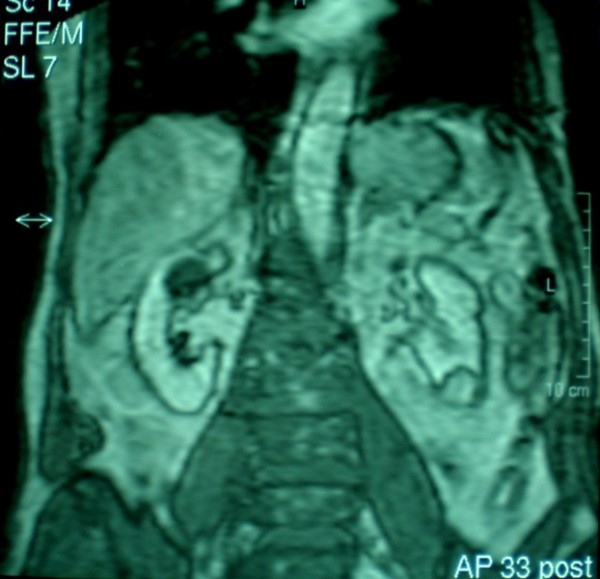
**Magnetic resonance imaging: An angiomyolipoma of the left lower lobe**.

The patient was a 70 year old asymptomatic male with a history of cholelithiasis in whom a solid 1,3 cm medial left renal mass was diagnosed incidentally by ultrasound. There was no significant past medical history, specifically seizures or mental retardation. On physical examination, head, neck, heart and lungs were normal. Neurological testing gave normal results. Blood renal tests were normal. Computed tomography (CT) showed a 1,3 cm well- defined, exophytic solid mass without cystic characteristics in the mid- portion of the left kidney, whereas on the lower pole of the same kidney, a 3,3 cm mass that contained a significant amount of fat was revealed. This mass was radiographically compatible to an angiomyolipoma. Invasion or infiltration into the perinephric fat, collecting system of vessels or regional lymphadenopathy and metastases were not encountered. On subsequent magnetic resonance imaging (MRI), the two renal masses were confirmed. In the mid- portion of the left kidney a 1,3 cm well- defined, homogenous mass was described, which appeared hypointense relatively to the renal cortex on T1- weighted images and isointense on T2- weighted images (Figure 1). No central scar was detected. Furthermore, the presence of an angiomyolipoma in the left lower lobe was confirmed (Figure 2). The right kidney was unremarkable radiographically. A working diagnosis of renal cell carcinoma was made. The patient underwent a left radical nephrectomy through left subcostal incision and recovered uneventfully.

### Pathologic Findings

#### Macroscopic examination

On gross examination, at the lower pole of the kidney an ovoid, yellowish 2,8 × 1,8 × 1,3 cm lesion which seemed to invade in the perinephric fat was found. In addition, there was a firm, well- encapsulated, brownish 1,7 × 1,2 × 1,0 cm tumor in the mid- portion of the kidney. Careful sectioning of the kidney did not reveal any additional lesion.

#### Microscopic examination

**Figure 3:**
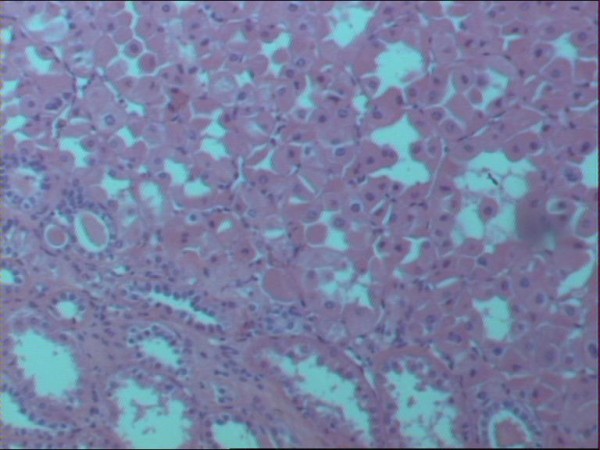
**Histopathological section of renal tumor, showing characteristic features of oncocytoma (haematoxylin- eosin ×100)**.

**Figure 4:**
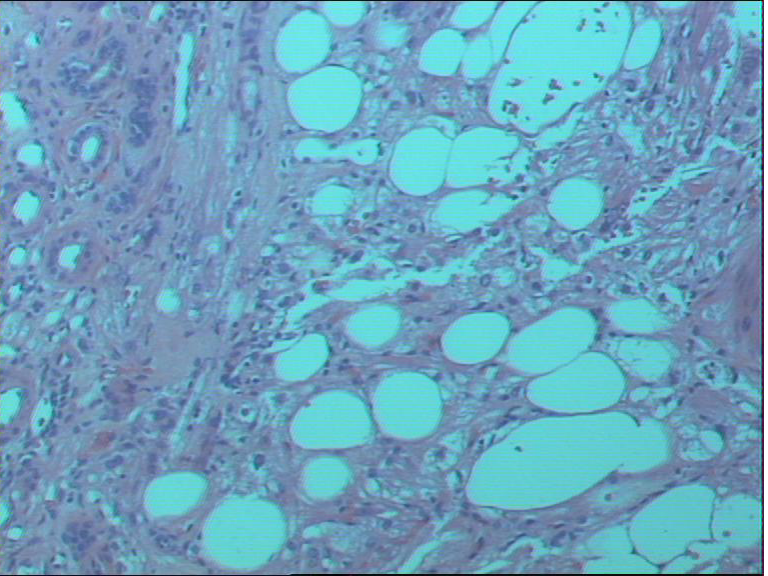
**Histopathological section of renal tumor, showing characteristic features of angiomyolipoma (haematoxylin- eosin ×100)**.

In the mid- portion of the kidney, the neoplasm exhibited a uniform population of plump cells arranged in alveolar- type nests and trabeculae with a granular, acidophilic cytoplasm. The morphological features were those of an oncocytoma (Figure 3). The lower pole tumor had features of an angiomyolipoma with predominant lipomatous and myomatous components. Thick- walled vessels and areas of hemorrhage within the tumor were present. Small foci of epithelioid cells without nuclear pleomorphism or increased mitotic activity were also noted (Figure 4).

Immunohistochemistry carried out on the sections of the oncocytoma showed negativity for antibody for vimentin and a weak positivity for antibody for CK 7.

The final confirmed diagnoses included an angiomyolipoma of the lower pole and oncocytoma of the mid- part of the left kidney.

## Discussion

Renal oncocytomas, first described by Zippel in 1942 [9], are benign, relatively non- frequent neoplasms, and they represent 3,2 to 5% of all primary renal masses arising from intercalating cells of the cortical collecting ducts. Oncocytoma is considered to be a benign neoplasm in the majority of cases; this is the reason why there is only one documented case of liver metastasis in literature [10]. However, it has also been reported that oncocytomas may occasionally involve fat tissue in up to 20% of cases and lymphovascular structures in up to 5% [11]. Most of these tumors are single and unilateral, although bilateral and multifocal cases have also been described [10]. Cytogenetically, there is not much evidence in the available literature, but it is possible to subdivide oncocytomas into three families, as far as their genetic abnormalities are concerned: oncocytomas with losses of chromosomes 1 or Y; oncocytomas with balanced translocations involving 11q13 (region encoding mitochondrial DNA) and oncocytomas with miscellaneous abnormalities [12]. Clinically, oncocytomas may be asymptomatic, but symptomatic patients may present with initial signs of haematuria, flank pain or palpable mass. The diagnosis of these benign lesions is generally achieved by computed tomography (CT) or magnetic resonance imaging (MRI). The appearance of a typical central stellate scar can occasionally be mimicked by necrosis in a renal cancer and this feature is not considered specific [13]. The characteristic histological features of oncocytomas are the dense eosinoplilic cytoplasm which may be exclusively or predominantly granular, generally uniform nuclei, and abundant mitochondria [10]. Considering the lack of diagnostic yield and low sensitivity of bioptical procedures and imaging techniques, it is well worth remarking that renal oncocytoma may overlap with other renal neoplasms, such as renal clear cell carcinoma with oncocytic features [14]. Therefore, surgical excision/nephrectomy remains the treatment of choice [15].

Renal angiomyolipomas are uncommon, benign tumor- like formations, consisting of mature adipose tissue, smooth muscle cells and thick- walled blood vessels and account for less than 1% of surgically removed tumors. The perivascular epithelioid cell is thought to be the cell of origin of angiomyolipomas [16].

A common immunophenotype of these tumors is the consistent expression of melanoma- associated antigens, particularly HMB- 45. Angiomyolipomas are commonly associated with tuberous sclerosis, which is an autosomal dominant disorder characterized by seizures, mental retardation, skin lesions and hamartomatous lesions in many organs [17]. Rarely, angiomyolipomas can be associated with von- Hippel Lindau disease, von Recklinghausen syndrome, and autosomal dominant polycystic kidney disease [18]. However, about 80% of renal angiomyolipomas occur sporadically and develop predominantly in women between the fourth and seventh decade of life, and are usually large, symptomatic, single and unilateral. In contrast, angiomyolipomas associated with tuberous sclerosis most often occur at a younger age and they tend to be asymptomatic, small, multifocal and bilateral [19]. Our patient, had no obvious clinical manifestations of tuberous sclerosis. Multiple renal angiomyolipomas, according to the criteria set forth by Gomez [20] are considered diagnostic of tuberous sclerosis complex. Meticulous examination of the nephrectomy specimen in our case, however, did not reveal additional foci of angiomyolipoma. The epithelium lining the cysts in tuberous sclerosis has been described as morphologically distinctive; that is, the lining cells are large and often strongly eosinophilic with large, hyperchromatic nuclei and usually identifiable mitoses [17]. Nevertheless, these features were not present in microscopic sections in our case. Therefore, we regarded the angiomyolipoma in our case as sporadic (non- tuberous sclerosis associated).

The diagnosis or renal angiomyolipoma is usually straight forward due to its characteristic sonographic appearance and its typical CT morphology. Ultrasonographically, most angiomyolipomas are hyperechogenic lesions resembling the echogenicity of the renal sinus fat; the fat component accounts for the negative attenuation values on CT scans and the hyperintensity on T1- weighted MR images [21]. However, these tumors can mimic a renal cell carcinoma, particularly if the fat content is low or if the fat is obscured by blood.

As the risk of haemorrhage in angiomyolipomas less than 4 cm in diameter is minor, the surgical treatment in asymptomatic cases is generally reserved for those tumors that are greater than 4 cm. Surgical excision, including radical nephrectomy, may remain the treatment of choice; nonetheless, in the appropriate setting, partial nephrectomy, enucleation, or wedge resection can be alternative options [21].

Although concurrence of renal cell carcinoma and oncocytoma within the same kidney is well recognized [19], the simultaneous unilateral existence of angiomyolipoma and oncocytoma is uncommon. To our knowledge, only 16 cases [1-3] of concurrent angiomyolipoma and oncocytoma, including 3 cases in association with tuberous sclerosis, have been reported in the literature, some as isolated case reports and others as collective series encompassing other tumors [1]. In cases where the relevant information was available, the age of the patients ranged from 42 to 73 years. Most cases occurred in women with only one case occurring in a man. This observation parallels the female predominance of angiomyolipoma as opposed to the male predominance of oncocytoma. Our patient was 70 years old, which fits in with this profile, but was a male, which makes our case remarkable.

The size of both tumors varied greatly and in some patients the presence of two tumors was not detected preoperatively [2]. In patients where the preoperative radiography was described, the presence of renal cell carcinoma could not be excluded. In our case, the angiomyolipoma measured 2, 8 cm and the oncocytoma was 1,7 cm. Both of them were revealed on abdominal imaging, but the radiologic evaluation could only clarify the type of the angiomyolipoma due to its characteristic radiologic features. However, the other lesion was felt to be a renal cell carcinoma. Therefore, a radical nephrectomy was indicated in our patient.

## Conclusion

In conclusion, we present an additional case of an oncocytoma associated with an angiomyolipoma, which is remarkable due to the fact that it was detected in a male patient. With respect to preoperative diagnosis, these neoplasms remain a challenge for the surgeon because they mimic renal cell carcinoma in the majority of cases. Our case did not occur in the setting of tuberous sclerosis. Of the 16 cases reported in the literature, only three cases associated with tuberous sclerosis complex have been documented. However, if angiomyolipomas are found incidentally in nephrectomy specimens along with other tumors, tuberous sclerosis should be excluded retrospectively.

## Consent

Written informed consent was obtained from the patient for publication of this case report and accompanying images. A copy of the written consent is available for review by the journal's Editor-in Chief.

## Competing interests

The authors declare that they have no competing interests.

## Authors' contributions

**TT **- responsible for critical revision of scientific content; **AY **- drafted the manuscript; **MK **- contributed substantially to manuscript conception and design; **GA **- assisted in the preparation of the manuscript; **EKV **- performed histopathological and immunohistochemical analyses and contributed to the pathology content; **ND **- have made substantial contributions to manuscript conception and acquisition of data; **IV **- the surgeon, approved the final version of the manuscript for publication; All authors read and approved the final version of the manuscript.
